# Spontaneous Ca^2+^ signals in the developing mammalian cochlea of live mice under different anaesthetic regimes

**DOI:** 10.1113/EP093267

**Published:** 2025-10-10

**Authors:** Francesca De Faveri, Walter Marcotti, Federico Ceriani

**Affiliations:** ^1^ School of Biosciences University of Sheffield Sheffield UK; ^2^ Neuroscience Institute University of Sheffield Sheffield UK

**Keywords:** afferent fibre, anaesthetic, development, hair cell, in vivo, isoflurane, ketamine, mouse, supporting cells

## Abstract

The pre‐hearing mouse cochlea undergoes critical periods of spontaneous Ca^2+^‐dependent activity that spreads across non‐sensory supporting cells and inner hair cells (IHCs). These signals have been shown to regulate not only the refinement of neural circuits along the auditory pathway towards functional maturity, but also the maturation of the hair cells into sensory receptors. Although the origin and interplay of these Ca^2+^ signals during cochlear development have recently been investigated in live mice, the impact of anaesthesia on in vivo functional measurements was not explored. Here, we investigate the effects of different anaesthetic regimes (ketamine and xylazine; 2.5% isoflurane; and 1.0%–1.5% isoflurane with the sedative acepromazine) that provided an effective unconsciousness to perform the surgery and Ca^2+^‐imaging recordings from the intact cochlea of live mice. The IHCs, supporting cells and spiral ganglion neuron terminals onto the IHCs showed spontaneous Ca^2+^‐dependent activity under all anaesthetic regimes, with a few significant differences observed between conditions. Calcium waves from supporting cells synchronized the activity of IHCs. Moreover, we found that the endocochlear potential, which is crucial for cochlear function, was unaffected by the different anaesthetics. However, low concentrations of isoflurane produced the most stable recordings of vital physiological signs in mice, including heart rate and breathing rate. Although all anaesthetic regimes tested appeared to be suitable for performing Ca^2+^ imaging from the cochlea of pre‐hearing live mice, a low concentration of isoflurane (1.0%–1.5%), combined with the pre‐anaesthetic sedative acepromazine and oxygenation, represents the most suitable approach to maintain a stable and long‐lasting depth of anaesthesia.

## Abstract (highlights)

- **What is the central question of this study?**
  How do different anaesthetic regimes impact spontaneous Ca^2+^ dynamics in hair cells, supporting cells and spiral ganglion neuron terminals in the cochlea of pre‐hearing mice?
- **What is the main finding and its importance?**
  The endocochlear potential, essential for normal cochlear function in vivo, was unaffected by anaesthesia at postnatal day 14. Both injectable (ketamine) and inhalant (isoflurane) anaesthetics preserved spontaneous Ca^2+^ signals in the cochlea, with a few significant differences observed between conditions. Low concentrations of isoflurane produced the most stable recordings of vital physiological signs in neonatal mice and, combined with acepromazine and oxygenation, represent the most suitable approach for recording cochlear spontaneous activity in pre‐hearing mice.

## 1 INTRODUCTION

Spontaneous Ca^2+^ activity has been shown to occur in the developing mammalian cochlea before external sensory input is detected (Kros et al., 1998). Over the years, the cellular mechanisms underpinning spontaneous Ca^2+^ activity in the cochlea have been investigated primarily using *ex vivo* cochlear preparations. These studies have shown that Ca^2+^ signals occur not only in inner hair cells (IHCs) (e.g. Beutner & Moser, 2001; Carlton et al., 2023; Johnson et al., 2011; Kros et al., 1998; Marcotti et al., 2003) but also in supporting cells (e.g. Tritsch et al., 2007; Wang et al., 2015). However, this *ex vivo* experimental approach has inherent limitations associated with the impossibility of replicating the complex anatomy, innervation and physiology of the mammalian cochlea, including the separation between perilymph and endolymph that impact on the function of both hair cells and supporting cells, the presence of an endocochlear potential and the efferent feedback from the brainstem (Nin et al., 2016; Nouvian et al., 2015; Vlajkovic & Thorne, 2022). Recent surgical and imaging advances have allowed the measurement of Ca^2+^ signals in the developing (De Faveri et al., 2025) and adult (Kim & Ricci, 2022, 2023) mouse cochlea in vivo. However, functional measurements in live mice require the use of anaesthetics (Navarro et al., 2021), which affects neurotransmission and thus physiological responses.

A variety of anaesthetic regimes are commonly used for auditory physiology measurements in rodents, each with specific advantages and limitations. The ketamine–xylazine mixture is widely used for auditory brainstem response (ABR) recordings in mice (e.g. Ingham et al., 2011), because it provides stable anaesthesia while maintaining low auditory thresholds (Cederholm et al., 2012; Ruebhausen et al., 2012). Nevertheless, the effects of ketamine–xylazine are relatively short lived, and the depth of anaesthesia can fluctuate during extended experimental procedures, requiring top‐up injections that might contribute to increased biological variability. Isoflurane delivered through inhalation is another anaesthetic commonly used in animal research, because it enables rapid induction and recovery, and continuous control of the depth of anaesthesia. However, high concentrations of isoflurane are known to depress neural activity and auditory responses (Cederholm et al., 2012; Ruebhausen et al., 2012; Stronks et al., 2010; Verdoodt et al., 2021). Although both ketamine–xylazine and isoflurane are anaesthetics widely applied in auditory studies in rodents, a systematic comparison of their effects on both spontaneous and evoked cochlear activity during development has not been explored.

In this study, we compared three different anaesthetic regimes that provided an effective unconsciousness to perform the surgery and Ca^2+^‐imaging recordings of spontaneous activity from the intact cochlea of live pre‐hearing mice. While keeping the mice under constant oxygenation, we compared the widely used anaesthetic mixture ketamine and xylazine with isoflurane (2.5%) or isoflurane (1.0%–1.5%) in combination with the subcutaneous injection of the pre‐anaesthetic sedative agent acepromazine (ACP) (Arras et al., 2001). We also investigated the depth of anaesthesia by measuring vital signs in pre‐hearing mice, such as heart rate and arterial oxygen saturation. Our study shows that the use of low concentrations of isoflurane with the pre‐anaesthetic sedative ACP is ideal for measuring cochlear function while preserving the in vivo physiological conditions of pre‐hearing mice.

## 2 MATERIALS AND METHODS

### 2.1 Ethics approval

The animal work was licensed by the UK Home Office under the Animals (Scientific Procedures) Act 1986 (PPL_PCC8E5E93) and approved by the University of Sheffield Ethical Review Committee (180626_Mar). Mice were kept in dedicated rooms with a 12h – 12h light–dark cycle, and both humidity and temperature were monitored continuously. For all in vivo experiments (auditory brainstem recordings, endocochlear potential and imaging experiments), mice were anaesthetized using different protocols: (1) inhalation of 2.5% isoflurane under oxygenation (0.8–1.0 L/min); (2) subcutaneous injection of acepromazine (ACP; 4 mg/kg body weight; ACP Injection 2 mg/mL, Elanco Animal Health UK Limited) 10 min before the delivery of isoflurane (1.0%–1.5%) under oxygenation (0.8–1.0 L/min); or (3) subcutaneous injection of ketamine (110–120 mg/kg body weight; Fort Dodge Animal Health, Fort Dodge, IA, USA) and xylazine (11–12 mg kg/body weight, Rompun 2%; Bayer HealthCare LLC, Tarrytown, NY, USA) with oxygenation (0.8–1.0 L/min). Note that in order to induce the depth of anaesthesia required for the non‐recovery surgery, the dose of ketamine had to be increased by 10%–20% in comparison to that normally used for non‐surgery in vivo experiments, such as ABR measurements (see below). At the end of each procedure, animals were killed by cervical dislocation followed by decapitation.

### 2.2 Mouse strains

Experiments were performed using *GCaMP6f* ^fl/fl^ mice (Jackson Laboratory, stock number 028865). To drive the expression of *GCaMP6f* in different cell types within the cochlear sensory epithelium, we used the following Cre lines. Hair cells were targeted using *Myo15‐Cre* mice (donated by Dr S. Safieddine), which drive Cre‐dependent recombination during early postnatal days (Caberlotto et al., 2011). *Pax2‐Cre* mice (donated by Dr A.K. Grove) allowed the investigation of Ca^2+^ signals in supporting cells and, to some extent, the hair cells (Ohyama & Groves, 2004). *NeuroD1‐Cre* mice (donated by Dr G. Pavlínková) were used to drive the expression of GCaMP6 in afferent fibres and terminals (Li et al., 2012). Recordings were performed in postnatal day (P)7–P8 *GCaMP6f^f^* ^l/fl^ *Myo15‐Cre* ^+/−^ and *GCaMP6f* ^fl/fl^ *NeuroD1‐Cre* ^+/−^ mice and in P7–P9 *GCaMP6f^f^* ^l/fl^ *Pax2‐Cre* ^+/−^ mice. All mice used were on the C57BL/6N background.

### 2.3 Assessment of vital parameters in live mice during anaesthesia

Anaesthetized C57BL/6N mice (P8–P11) were placed on a heated mat. A throat sensor connected to MouseOx Plus Pulse Oximeter (STARR Life Sciences Corp., USA) was placed on the mouse throat to record breathing rate, heart rate, pulse distension and arterial oxygen saturation over a 1 h period.

### 2.4 Surgery for in vivo cochlear experiments

Anaesthetized mice were positioned on a heating mat (belly facing upwards) to maintain a stable body temperature. The surgical procedure used for the in vivo imaging and endocochlear potential measurements has been described previously (De Faveri et al., 2025). In brief, access to the cochlea was obtained by cutting the skin, followed by the gentle separation of muscles and connective tissue on the side of the trachea. After exposing the apical coil of the cochlea, a small part of the bone covering the 8–18 kHz region in hearing mice (Müller et al., 2005) was gently removed with fine forceps. The surgical procedure was performed without breaking the lateral wall membranes sealing the cochlear partition, which prevented any disruption or mixing of the endolymph and perilymph solutions in the different cochlear compartments. The space above the cochlea was then filled with an extracellular solution at 37°C (mM: 135 NaCl, 5.8 KCl, 1.3 CaCl_2_, 0.9 MgCl_2_, 0.7 NaH_2_PO_4_, 5.6 d‐glucose and 10 Hepes–NaOH), which allowed the visualization of the cochlear sensory epithelium with water‐immersion objectives.

### 2.5 Endocochlear potential measurements

Borosilicate glass microelectrodes were filled with 150 mM KCl and mounted on a patch‐pipette holder attached to a micromanipulator. Following the same procedure described for the in vivo surgery, the microelectrode was inserted into the scala media through the spiral ligament and the stria vascularis to measure the endocochlear potential (EP) from C57BL/6N mice (P12). The ground electrode was inserted in the lateral muscles of the neck region. EP responses were recorded in current‐clamp mode (gapfree) using an Axopatch 200B amplifier (Molecular Probes, USA). Data acquisition was controlled using pClamp v.10 software and a Digidata 1440A (Molecular Devices, USA). Recordings were low‐pass filtered at 1.0 kHz (eight‐pole Bessel), sampled at 10 kHz and stored on a computer for off‐line analysis (Clampfit, Molecular Devices).

### 2.6 Auditory brainstem responses

After the induction of anaesthesia using the different regimes described above, the loss of the withdrawal reflex in response to a toe pinch was used to confirm that an adequate depth of anaesthesia had been reached. Constant oxygenation (0.8–1.0 L/min) was maintained throughout the recording. Mice were then placed onto a heat mat (37°C) in a sound‐proof chamber (MAC‐3 acoustic chamber, IAC Acoustic, UK). Subdermal electrodes were placed under the skin behind the pinna of each ear (reference and ground electrode) and on the vertex of the mouse head (active electrode) as previously described (Ingham et al., 2011). ABRs were recorded from male and female P29–P33 mice from the strains listed above. Sound stimuli were delivered to the ear by calibrated loudspeakers (MF1‐S, Multi Field Speaker, Tucker‐Davis Technologies, USA) placed 10 cm from the animal's pinna. Sound pressure was calibrated with a low‐noise microphone probe system (ER10B+, Etymotic, USA). Experiments were performed using a customized software package (Ingham et al., 2011) driving an RZ6 auditory processor (Tucker–Davis Technologies). Response thresholds were estimated from the resulting ABR waveform and defined as the lowest sound level at which any recognizable feature of the waveform was visible. Responses were measured for clicks and stimulus pure tones of frequencies at 3, 6, 12, 18, 24, 30, 36 and 42 kHz. Stimulus sound pressure levels (SPLs) were typically 0–95 dB SPL, presented in steps of 5 dB SPL. The brainstem response signal was averaged over 256 repetitions. Tone bursts were 5 ms in duration with a 1 ms on–off ramp time, which was presented at a rate of 42.6 bursts/s.

Wave 1 amplitude and latency were measured from ABR recordings obtained by stimulating mice with a pure tone (12 kHz) using custom software (Ceriani, 2024). We selected the value of 12 kHz because it is close to the frequency range used for the in vivo work. Automatic identification was reviewed manually and, if required, adjusted to the correct peak. The wave 1 amplitude was calculated as the difference between the amplitude of the first peak and the first trough of the ABR waveform; the latency was calculated as the delay of the wave 1 peak from the beginning of the recording. Given that the distance of the speaker from the animal is 10 cm (see above), this leads to a delay in the signal of ∼0.3 ms.

### 2.7 Two‐photon imaging

After the surgical procedure, the anaesthetized mouse was transferred to the stage of a two‐photon laser‐scanning microscope (Bergamo II System B232, Thorlabs Inc., USA), equipped with a mode‐locked laser system operating at 925 nm, 80 MHz pulse repetition rate and <100 fs pulse width (Mai Tai, Spectra‐Physics, USA). Images were acquired with a field size of 1024 pixel width and variable pixel height. The magnification of the microscope was adjusted for each experiment to contain the maximal area at the same focal plane in the field of view. The cochlea was localized and centred through the eyepieces using a low‐magnification objective. This lens was then switched to a higher‐magnification, water‐immersion objective (CFI75 LWD 16X W, NA 0.8; CFI75 Apochromat 25XC W, NA 1.1, Nikon), which was lowered slowly towards the surgical opening filled with extracellular solution. The cochlea was localized through the imaging software (Thorlabs Inc., USA) using landmarks produced by the autofluorescence of the surrounding tissue and opening in the bone. Imaging recordings were normally performed for ≤1 h before the mouse was killed by a Schedule 1 method. Comparisons between different anaesthetic regimes in this and our previous study (De Faveri et al., 2025) were performed using the same mouse lines, imaging set‐up and acquisition settings. For all experimental conditions, the focal plane was set at the level of the IHC nuclear region.

### 2.8 Image analysis

Images recorded with the two‐photon system were saved on an external large‐capacity storage for off‐line processing. The image analysis consisted of the following steps. First, recordings were examined visually using ImageJ (NIH) to identify and remove any frame interval that was affected by excessive drifts preventing signal detection. Second, recordings were filtered with a three‐dimensional Gaussian filter (2 × 2 × 2 pixels) to remove noise and improve signal detection. Third, using custom python routines and Graphical User Interface (GUI), intervals of frames during which the preparation was out of focus (e.g. owing to breathing by the mouse) were identified and removed from the recording. This was performed using a previously described semi‐automated procedure (De Faveri et al., 2025). Frames marked for removal were replaced by the last available in‐focus frame to preserve the timing of the recording. In the fluorescence traces, these ‘missing’ time points were replaced by a linear interpolation of the nearest fluorescence values. Fourth, after removing out‐of‐focus frames, each movie was adjusted for lateral drift using the NoRMCorre algorithm from the CaImAn package (Pnevmatikakis & Giovannucci, 2017). The pixel values of the motion‐corrected movies were adjusted to ensure that the average was the same as the original recording. For each type of experiment, a semi‐automatic procedure was devized to segment the field of view in regions of interest (ROIs) and extract the fluorescence signal. Movies were opened in the Napari image software (Sofroniew et al., 2025) equipped with custom plugins. The ROIs and annotations were generated as ‘label’ layers in Napari. For the investigation of the IHC activity using *GCaMP6f* ^fl/fl^ *Myo15‐Cre* ^+/−^ mice, we first created an average image of the entire image stack. This was then fed to a Cellpose (Stringer et al., 2021) algorithm (*cyto2* model) to automatically generate a mask of ROIs for bright objects in the field of view. The masks were eroded by one to three pixels to minimize signal contamination between adjacent cells and adjusted manually by the experimenter when required (e.g. to split merged cells or to join split ROIs). The masks of subsequent recordings of the same field of view were examined to ensure manually that the same IHCs were tagged with a unique label across recordings. This procedure allowed fluorescence traces to be stitched across several recordings. Different recordings were aligned using landmarks present in the field of view, such as bright supporting cells or a ‘double’ row of IHCs. Fluorescence traces for each ROI were extracted by calculating the average intensity of the pixels comprising an ROI for each frame, and the relative change of fluorescence intensity compared to baseline (d*F*/*F*_0_) was used for further analysis. For the analysis of supporting cell activity (*GCaMP6f* ^fl/fl^ *Pax2‐Cre* ^+/−^ mice), we initially drew a polygonal line across the location of the IHCs using an average image of the entire stack as reference. This allowed the calculation of the position and orientation of the Ca^2+^ waves in comparison to the IHC position, and their longitudinal and radial extensions. Each image of the stack was then binned by a factor of two and manually thresholded. We used the Voronoi‐Otsu labelling algorithm to semi‐automatically generate three‐dimensional ROIs that represented a Ca^2+^ wave across different frames. These three‐dimensional ROIs were revised manually in Napari to merge and split events and remove artefacts. Events that involved Ca^2+^ transients in individual isolated cells were excluded from the analysis. Recordings were then manually revised and bidimensional ROIs manually drawn across the maximal extension of Ca^2+^ waves that were not selected by the preceding automatic method or for which automatic detection was not satisfactory. These Ca^2+^ waves were included in the calculation of the frequency and maximal extension of spontaneous events, but excluded from the calculation of their dynamical properties (e.g. expansion speed). Repetitive imaging recordings of the same field of view were collated and considered as one recording. For spiral ganglion neuron (SGN) synaptic terminal activity (*GCaMP6f* ^fl/fl^ *NeuroD1‐Cre* mice), images were averaged, filtered with a white tophat filter with a radius of 10–20 pixels, then segmented using the Voronoi‐Otsu labelling algorithm. The labels produced by this algorithm were inspected visually, and ROIs were adjusted manually using Napari. A custom‐trained Cellpose model was used to semi‐automatically label IHC bodies in the average image. Based on their proximity to the identified hair cells, SGN terminals were ‘assigned’ to a specific IHC. For SGN terminals that were ‘in between’ hair cells or for which an IHC could not be assigned unequivocally, we compared their activities with the two closest IHCs by calculating the correlation coefficient between the fluorescence traces and automatically assigned the SGN terminals to the IHC displaying the highest correlation. SGN terminal labels were further annotated as modiolar, pillar or ‘middle’, depending on their position around the IHC body. All labels were inspected and adjusted manually. Fluorescence traces for each ROI were extracted by calculating the average intensity of its pixels for each frame, and d*F*/*F*_0_ was used for further analysis.

### 2.9 Calcium event detection

A custom GUI (De Faveri et al., 2025) was used to inspect the traces and select ‘peaks’ corresponding to Ca^2+^ transients in individual IHCs or SGN terminals. Peaks were initially selected based on a prominence criterion (*find_peaks* function of *scipy* Python module). The resulting identification was revised manually to correct artefacts and add missing events. Only peaks that had an amplitude of >2SD of a trace and a full duration at half‐maximum of >200 ms were considered to be genuine. For IHCs, when the same field of view was recorded multiple times, we combined the peaks identified in subsequent recordings. Frequencies were computed as the total number of peaks divided by the total duration of the combined recordings. An ‘event’ was defined as a Ca^2+^ transient in one or multiple IHCs in the field of view. The IHCs in the field of view were assigned to the same event if they had a peak within 2 s of one another. We considered ‘single’ events that involved only one IHC and ‘multiple’ events that involved three IHCs or more. A ‘multiple’ event that involved IHCs with a gap of more than four non‐participating cells was considered as two separate events. This value was determined by observation of fluorescence movies. Pearson correlation coefficients were calculated between the traces of every pair of IHCs in the field of view, provided that the two cells had ≥5 min of simultaneous recording (this was not always the case for repeated recordings of the same field of view, where some cells were not consistently in focus). Before calculating the correlation coefficient, traces were detrended by subtracting a rolling median. Averages and SDs of correlation coefficients *r*_s_ were calculated using the Fisher's *z*‐transformation: *z* = arctanh(*r*_s_); avg(*r*_s_) = tanh[avg(*z*)]; SD(*r*_s_) = tanh[SD(*z*)]. The full duration at half‐maximum of Ca^2+^ waves was calculated by extracting a pixel‐average trace of the Ca^2+^ wave around its maximum. The frequency of Ca^2+^ waves was calculated as the number of events divided by the total duration of the recording. For SGN terminals, an ‘IHC’ trace was generated by averaging the traces of all its associated SGN terminals. For SGN terminals, we identified as an ‘event’ a Ca^2+^ transient in one or multiple SGN terminals associated with an IHC. SGN terminals contacting the same IHC were assigned to the same event if they had a peak within 1 s of one another. Frequency quantification and cross‐correlation analysis were performed for recordings that lasted ≥5 min. For quantification of other properties of Ca^2+^ signals, shorter recordings were also included in the analysis.

### 2.10 Statistical analysis

For multiple comparisons, one‐way ANOVA followed by a suitable *post hoc* test was used for normally distributed data; otherwise, Kruskal–Wallis with Dunn's *post hoc* test was used. A value of *p* < 0.05 was selected as the criterion for statistical significance. Statistical comparisons of the linear fits were performed using Student's two‐tailed *t*‐tests, considering the estimated covariance matrix, with the Bonferroni *post hoc* test. Statistical tests on correlation coefficient data were done after Fisher's *z*‐transformation (see above). Only mean values with a similar variance between groups were compared. Average values are quoted in text and figures as the mean ± SD. Animals of either sex were assigned randomly to the different experimental groups. No statistical methods were used to define sample size, which was defined based on previously published similar work from our laboratory. Animals were taken from several cages and breeding pairs over a period of several months.

## 3 RESULTS

To investigate the effect of different anaesthetic regimes on cochlear function in vivo, we combined a recently described surgical procedure with the use of mice expressing GCaMP6f in the IHCs, their afferent terminals or surrounding supporting cells (De Faveri et al., 2025). This in vivo approach prevented any disruption or mixing of the endolymph and perilymph solutions within the cochlear partition. However, the potential confounding effect of the anaesthetic regime on cochlear function in vivo was not explored fully (De Faveri et al., 2025). Therefore, we compared the dynamics of spontaneous activity in mice anaesthetized with either 2.5% isoflurane (De Faveri et al., 2025), a ketamine and xylazine mixture, or with a lower concentration of isoflurane (1.0% and 1.5%) delivered in combination with the pre‐anaesthetic sedative agent ACP (Arras et al., 2001). In all conditions, mice were kept under constant oxygenation (0.8–1.0 L/min).

### 3.1 Effect of the different anaesthetic regimes on auditory function

**FIGURE 1:**
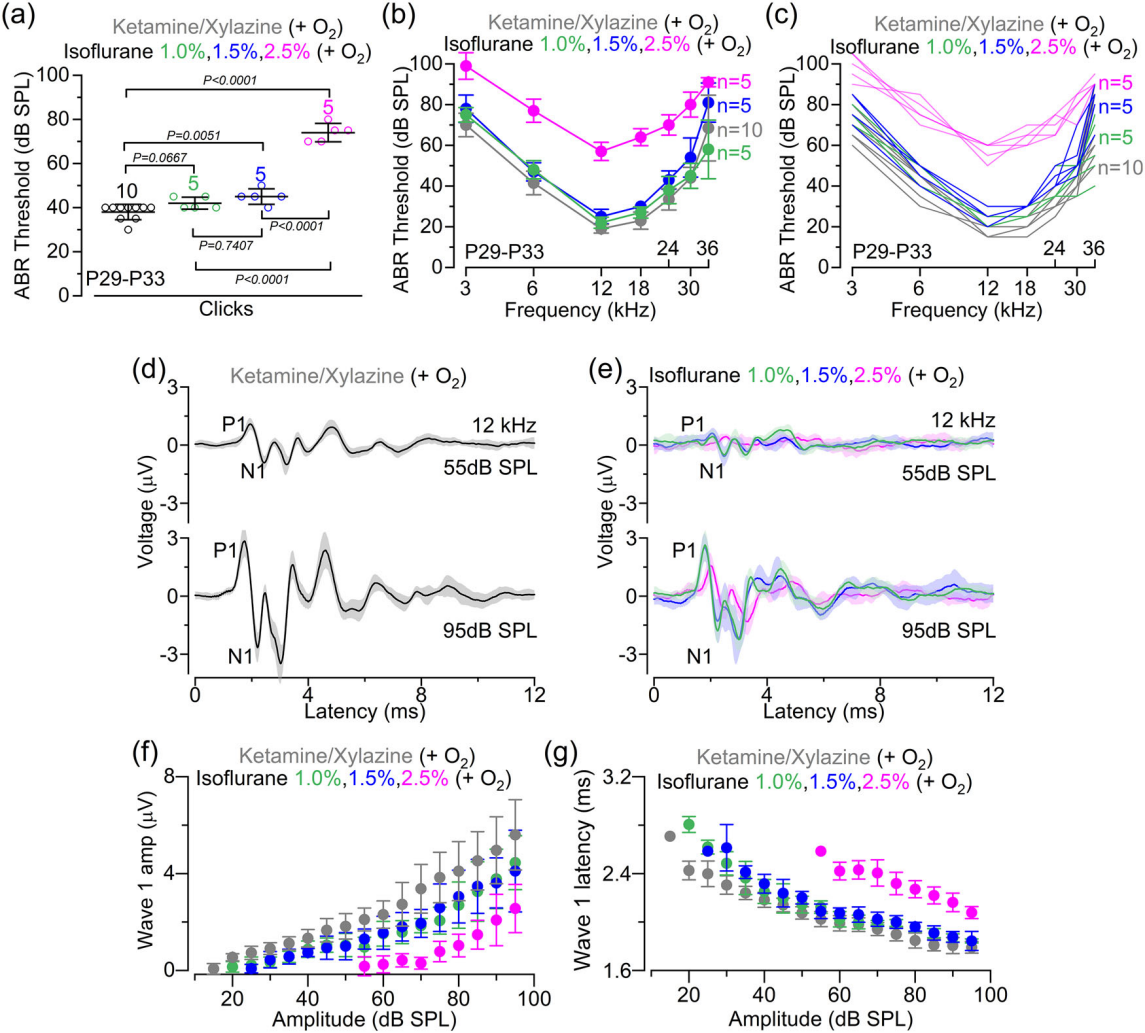
Effect of anaesthesia on ABRs of adult mice. (a) ABR thresholds elicited by click acoustic stimuli applied to C57BL/6N mice recorded under the different anaesthetic regimes under oxygenation (0.8–1.0 L/min): ketamine and xylazine (P29–P33, 10 mice), isoflurane 2.5% (P30, 5 mice), ACP + isoflurane 1.5% (P30, 5 mice), ACP + isoflurane 1.0% (P30, 5 mice). Average data are shown superimposed to single data points plotted as open circles. Significance values are from one‐way ANOVA followed by Tukey's *post hoc* test. (b, c) ABR thresholds for frequency‐specific pure tone stimulations ranging from 3 to 36 kHz recorded from the four experimental conditions described above. The number of mice used for each anaesthetic condition is as mentioned above. Statistical comparisons between the different conditions were obtained from two‐way ANOVA followed by Tukey's *post hoc* test: *p* = 0.5233 (ketamine and xylazine vs. isoflurane 1.0%); *p* = 0.0007 (isoflurane 1.0% vs. isoflurane 1.5%); and *p* < 0.0001 (all other comparisons). (d,e), Average ABR waveform responses at 12 kHz at increasing stimulus intensity (in decibels sound pressure level) using ketamine–xylazine (d) and the different concentrations of isoflurane (e) obtained from the same mice listed in panels (a–c). Continuous lines represent average values and the shaded areas the SDs. P1 and N1 indicate the positive and negative peaks of wave 1, respectively. (f, g) Average amplitude [(f) size of wave 1 between P1 and N1 as indicated in panels (d, e): left panel] and latency [(g) time between the onset of the stimulus and P1: right panel] as a function of the sound intensity recorded under the different anaesthetic regimes. Statistical comparisons between the different conditions were obtained from two‐way ANOVA followed by Tukey's *post hoc* test: wave 1 amplitude, *p* = 0.9865 (isoflurane 1.0% vs. isoflurane 1.5%) and *p* < 0.0001 for all other comparisons; wave 1 latency, *p* = 0.7366 (isoflurane 1.0% vs. isoflurane 1.5%) and *p* < 0.0001 for all other comparisons. All average data are reported as the mean ± SD. Abbreviations: ABR, auditory brainstem response; ACP, acepromazine; P, postnatal day.

Considering that anaesthetics act primarily by affecting physiological responses in the CNS (Hao et al., 2020; Platholi & Hemmings, 2022), we initially compared the impact of the above selected anaesthetic regimes on hearing function, which was assessed using ABRs. Using the ketamine and xylazine mixture, which is the preferred choice for hearing function measurements, ABRs had thresholds (Figure 1a–c) comparable to those we have reported previously (Underhill et al., 2025). Thresholds for both clicks and pure tone stimuli in ketamine and xylazine mixture were significantly lower compared with those recorded in the presence of ACP + isoflurane 1.5% and 2.5%, but not with ACP + isoflurane 1.0% (Figure 1a–c). To assess the effects of the different anaesthetic regimes on the sound‐induced output of the cochlea, we analysed ABR wave 1, which is generated by the summed response to sound of the afferent nerve fibres innervating the IHCs (Møller & Jannetta, 1982; Schaette & McAlpine, 2011). ABR wave 1 was analysed for the 12 kHz responses (Figure 1d, e), because this closely matches the cochlear region used for the in vivo cochlear experiments. We found that wave 1 amplitude (Figure 1f) and latency (Figure 1g) were significantly reduced in the presence of isoflurane compared with ketamine (*p *< 0.0001 for all comparisons, two‐way ANOVA). However, both wave 1 amplitude and latency were greatly improved when the concentration of isoflurane was decreased from 2.5% to either 1.0% or 1.5% (Figure 1f, g; *p* < 0.0001 for both comparisons, two‐way ANOVA), reaching values that more closely matched those in ketamine. These results show that apart from a relatively small reduction in wave 1 amplitude and latency in comparison to those recorded with the commonly used ketamine, low doses of isoflurane with the addition of ACP are suitable for ABR recordings.

### 3.2 Physiological parameters in mice under anaesthesia

**FIGURE 2:**
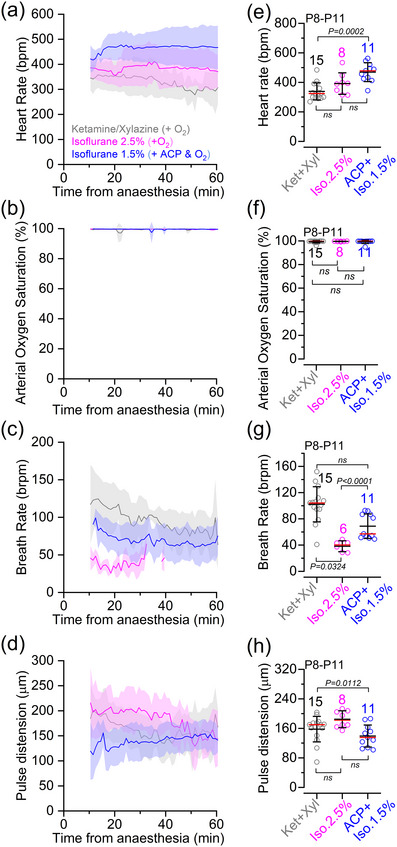
Physiological parameters in pre‐hearing anaesthetized mice. (a–d) Average heart rate (a), arterial oxygen saturation (b), breathing rate (c) and pulse distension (d) recorded from pre‐hearing mice (P8–P11) under different anaesthetic regimes shown in panel (a). Recordings were performed over a 1 h period, which corresponds to the typical duration of the experiments investigating Ca^2+^ signalling in the cochlea in anaesthetized mice (Figures 3, 4, 5, 6). Note that recordings started about 10 min after the induction of anaesthesia. Also note that when using 2.5% isoflurane, the breathing rate generally fell below the detection limit of the equipment (25 breaths/min) after ∼30 min in most of the recordings (c). (e–h) Average values of the different physiological parameters shown in panels (a–d), respectively, during the full duration of the recordings. Average data: mean ± SD [red lines in panels (e–h) represent the median]. Number of mice for each physiological measurement is shown above the data in panels (e–h). Statistical values in panels (e–h) are from Kruskal–Wallis test followed by Dunn's *post hoc* test. Abbreviations: ABR, auditory brainstem response; ACP, acepromazine; Iso, isofluorane; Ket, ketamine; P, postnatal day; Xyl, xylazine.

To assess the general physiological status of pre‐hearing mice (P8–P11) under deep anaesthesia and the time‐dependent stability of different anaesthesia protocols, we monitored various vital parameters, such as arterial saturation, heart rate, breathing rate and pulse distension (Figure 2a–h). This was achieved by placing the pulse oximeter sensor at the throat region of pre‐hearing mice (see Materials and Methods). These measurements were taken over a 1 h period, which corresponds to the typical duration of the in vivo cochlear experiments shown below. The heart rate was found to be stable throughout the recordings when using isoflurane, but declined over time with ketamine and xylazine, making it significantly reduced compared with 1.5% isoflurane +CP (Figure 2a, e). Values under 1.5% isoflurane +ACP administration were also closer to the values measured in age‐matched unanaesthetized mice (∼600 beats/min; Sato, 2008). Arterial oxygen saturation, which provides an indication of the level of oxygen in the blood, was >98% in all three different anaesthetic regimes (Figure 2b, f). Respiratory function, assessed by the number of breaths per minute, was comparable between ketamine–xylazine and 1.5% isoflurane + ACP (Figure 2c, g). In the presence of 2.5% isoflurane, the breathing rate dropped below the detection limit of the equipment for most mice after ∼30 min from the start of anaesthesia (Figure 2c, g). Despite this technical limitation, all mice under 2.5% isoflurane showed visible breathing movements and a measurable heart rate throughout the duration of the experiments. Unsurprisingly, the breathing rate was depressed in all anaesthetic conditions compared with unanaesthetized mice (∼250 breaths/min in the second postnatal week; Chen et al., 2013; Zehendner et al., 2013), consistent with the known suppressive effects of anaesthetics on respiratory function. Finally, we measured the pulse distension of the arteries at the neck region, which provides additional information about the cardiovascular and respiratory status of the mice under anaesthesia. We found that pulse distension was slightly reduced in 1.5% isoflurane + ACP in comparison to ketamine and xylazine (Figure 2d, h). These results indicate that all anaesthesia protocols were able to maintain several vital physiological parameters in pre‐hearing mice.

### 3.3 Endocochlear potential in different anaesthetic regimes

A key aspect of cochlea function in vivo is the presence of the EP, which is a positive potential of about +80 mV between the endolymph and perilymph (Von Bekesy, 1952). The EP contributes greatly to the electrical driving force for the mechanoelectrical transduction current and normal hair cell physiology (Fettiplace & Kim, 2014; Johnson et al., 2012). In mice, the EP develops from about the end of the first postnatal week and reaches about +60 mV (66%–75% of its adult value) by P14 (Li et al., 2020). We found that the EP was not significantly different between the three anaesthetic regimes (ketamine and xylazine, 47 ± 12 mV; 2.5% isoflurane, 39 ± 5 mV; and 1.5% isoflurane + ACP, 38 ± 2 mV; five mice for each condition, P12, *p* = 0.1433, one‐way ANOVA) and was comparable to that previously reported for aged‐matched mice (Li et al., 2020). Having established that the gross cochlear physiology is not affected by the different anaesthetics at P12 or in pre‐hearing mice (De Faveri et al., 2025), we investigated spontaneous Ca^2+^ dynamics in the hair cells, supporting cells and afferent terminals.

### 3.4 in vivo spontaneous Ca^2+^ signals in pre‐hearing IHCs

**FIGURE 3:**
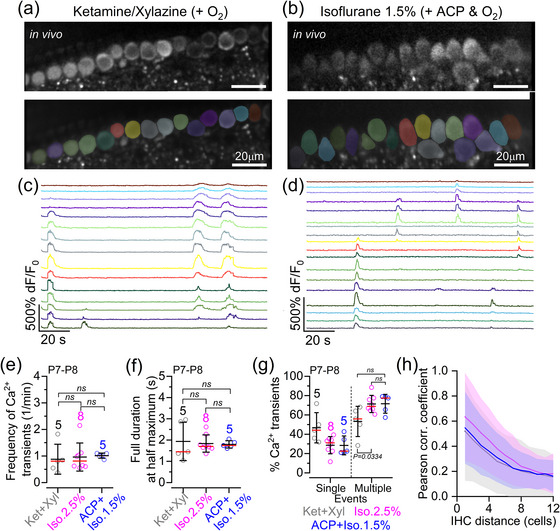
In vivo spontaneous Ca^2+^ signals in cochlear inner hair cells of pre‐hearing mice under different anaesthetic regimes. (a, b) Spontaneous Ca^2+^ signals recorded from live P8 *GCaMP6f* ^fl/fl^ *Myo15‐Cre* ^+/−^ mice anaesthetized using either ketamine and xylazine (a) or ACP + 1.5% isoflurane (b). Top panels show average‐intensity projections of a time‐lapse recording highlighting GCaMP6f expression in apical‐coil IHCs. Bottom panels show ROIs generated using a semi‐automated identification approach (see Materials and Methods), which were used to measure spontaneous Ca^2+^ transients from individual IHCs from the images depicted in the top panels. (c, d) Fluorescence time series computed as pixel averages from the ROIs in panels (a, b), respectively, highlighting spontaneous Ca^2+^ activity in IHCs. Note that colours are matched to panels (a, b), respectively. (e) Frequency of spontaneous Ca^2+^ transients in IHCs of P7–P8 *GCaMP6f* ^fl/fl^ *Myo15‐Cre* ^+/−^ mice as a function of anaesthetic regime (see Materials and Methods). Averages were calculated from individual mice: five mice (128 IHCs, ketamine and xylazine), eight mice (278 IHCs, 2.5% isoflurane) and five mice (151 IHCs, ACP + 1.5% isoflurane). All data were from IHCs that had ≥5 min of total recording time. In this and the following figures, the data used for the 2.5% isoflurane are from De Faveri et al. (2025). (f) Full duration at half‐maximum of Ca^2+^ transients as a function of anaesthetic regime recorded from the same set of data used for panel (e). (g) Percentage of events where Ca^2+^ transients were simultaneously present in a ‘single’ or ‘multiple’ (more than two) IHCs. Note that two‐cell events (14.3%) were excluded to minimize any misrepresentation of random coincidental events. Averages were calculated from individual mice: five mice with 126 active IHCs out of 128 (ketamine and xylazine); eight mice with 276 active IHCs out of 278 (ACP + 2.5% isoflurane); and five mice with 154 active IHCs out of 154 (ACP + 1.5% isoflurane). Average data in panels (e–g) are shown as mean ± SD (median: red lines). Statistical results were from Kruskal–Wallis (e, f) and one‐way ANOVA, Tukey's multiple comparison test (g). (h) Average correlation coefficient (continuous line, average Pearson correlation coefficient; shaded area, SD) as a function of the distance between IHCs. Abbreviations: ABR, auditory brainstem response; ACP, acepromazine; d*F*/*F*_0_, relative change of fluorescence intensity compared to baseline; IHC, inner hair cell; Iso, isoflurane; Ket, ketamine; P, postnatal day; ROI, region of interest; Xyl, xylazine.

Calcium signals in pre‐hearing IHCs (P7–P8) were studied using *GCaMP6f* ^fl/fl^ *Myo15‐Cre* mice (see Materials and Methods). In in vivo experimental conditions, IHCs exhibited spontaneous Ca^2+^ transients under all anaesthetic regimes tested (Figure 3a–h). The average frequency of Ca^2+^ transients in P7–P8 mice was not significantly different across the anaesthetic regimes (Figure 3e; *p* = 0.3510, Kruskal–Wallis test). Similar results were obtained for the full duration at half‐maximum of Ca^2+^ transients (Figure 3f; *p* = 0.8865). In all anaesthetic conditions, the majority of Ca^2+^ transients observed in P7–P8 IHCs occurred almost simultaneously in more than three neighbouring cells, although the fraction of coordinated transients seemed to be increased slightly in the presence of 2.5% isoflurane compared with ketamine–xylazine (Figure 3g; *p* = 0.0334, Dunn's *post hoc* test). This indicates that the proportion of Ca^2+^ transients in IHCs that occurred as spontaneous uncoordinated events independently from neighbouring cells was higher with the injectable anaesthetic (Figure 3g). Nevertheless, these findings further support the existence of intrinsically generated Ca^2+^ transients in immature IHCs, which has been debated based on previous recordings from cochlear explants (Johnson et al., 2017, 2011; Sendin et al., 2014; Tritsch et al., 2007; Wang et al., 2015). We showed previously that during coordinated Ca^2+^ transient activity, on average, about seven IHCs were recruited when using 2.5% isoflurane, which resulted in a high degree of correlation between the activity of nearby IHCs, decreasing exponentially with the distance between cells (De Faveri et al., 2025). A similar exponential decrease was also observed in the presence of ketamine and 1.5% isoflurane (Figure 3h).

### 3.5 Spontaneous calcium waves in developing supporting cells from live mice

In the developing cochlear sensory epithelium, the supporting cells spontaneously release ATP in physiological conditions (Anselmi et al., 2008; Tritsch et al., 2007; Wangemann, 1996). The binding of extracellular ATP to G‐protein‐coupled P2Y autoreceptors located on the supporting cells (Babola et al., 2021; Hool et al., 2023; Jagger & Forge, 2015) leads to the activation of phospholipase C and the production of IP_3_, which then induces the release of Ca^2+^ from the endoplasmic reticulum into the cytosol through the activation of IP_3_ receptors and additional release of ATP (Beltramello et al., 2005; Ceriani et al., 2025; Piazza et al., 2007). This cascade of events enables the propagation of regenerative intercellular Ca^2+^ waves in cochlear supporting cells that have been shown to modulate Ca^2+^ action potentials in developing IHCs in *ex vivo* cochlear explants (Johnson et al., 2017; Wang et al., 2015). More recently, Ca^2+^ waves have also been examined in live P7–P9 pre‐hearing mice using 2.5% isoflurane (De Faveri et al., 2025). We investigated, therefore, whether using different anaesthetic regime would affect Ca^2+^ wave dynamics. As for the IHCs (Figure 3), we tested ketamine–xylazine and two concentrations of isoflurane (1.0% and 1.5% + ACP). Given that we obtained similar results between 1.0% and 1.5% isoflurane, the data between the two sets of experiments were combined.

**FIGURE 4:**
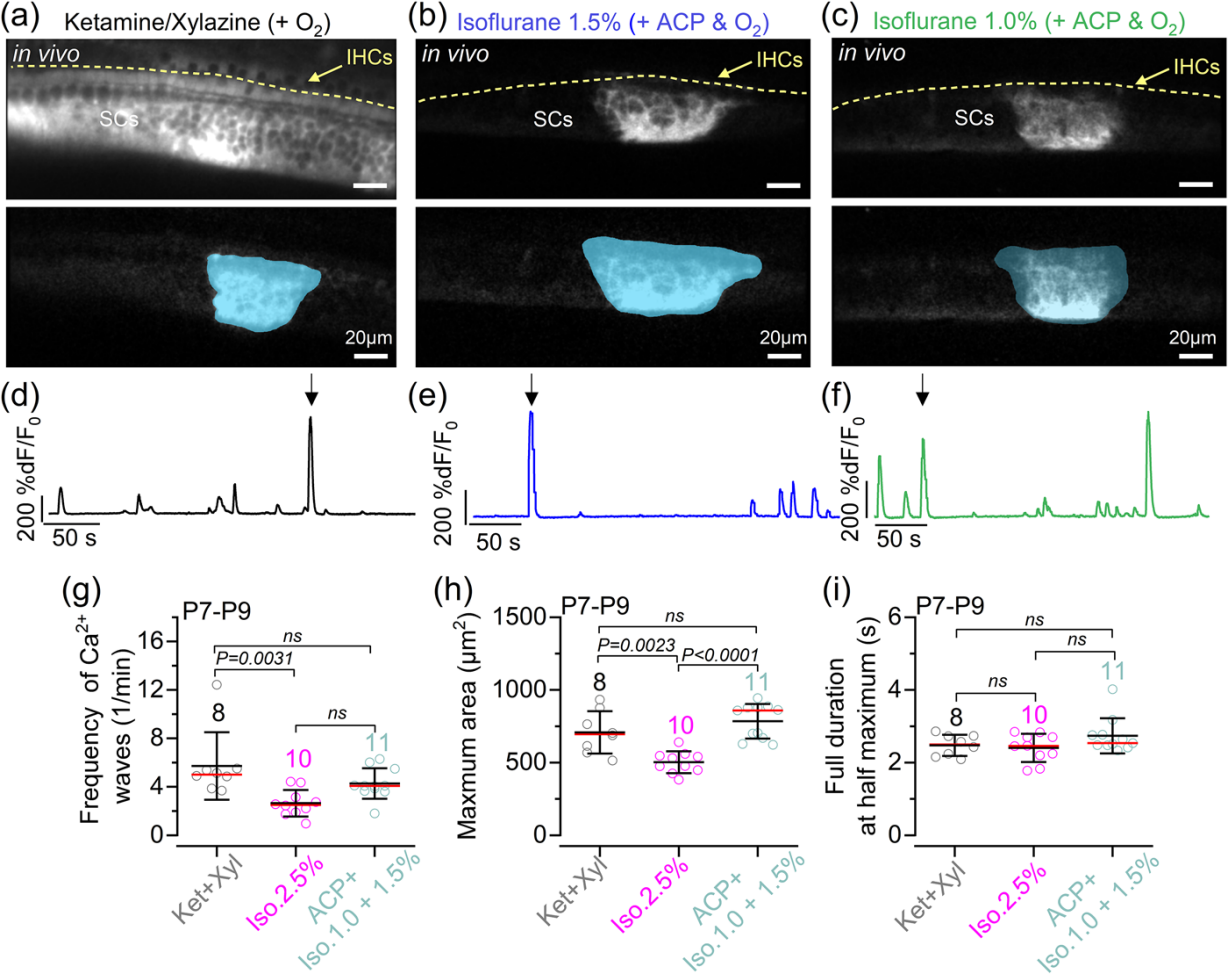
Calcium waves in supporting cells in live pre‐hearing mice under different anaesthetic regimes. (a–c) Average intensity projections (top panels) displaying GCaMP6f expression in the cochlear epithelium from *GCaMP6f* ^fl/fl^ *Pax2‐Cre* ^+/−^ mice at P8 under three different anaesthetic regimes and constant oxygenation [(a) ketamine and xylazine; (b) ACP + 1.5% isoflurane; and (c) ACP + 1.0% isoflurane]. Bottom panels show examples of individual Ca^2+^ waves in the three different anaesthetic regimes with the semi‐automatic ROI segmentation superimposed. (d–f) Fluorescence trace from ROIs drawn in panels (a–c), respectively. (g) Average frequency of Ca^2+^ waves recorded from P7–P9 mice (Ca^2+^ waves per minute). Each data point represents the average of the recordings obtained from each of the 29 mice: ketamine and xylazine, 8 mice; 2.5% isoflurane, 10 mice; 1.0% or 1.5% isoflurane + ACP, 11 mice. Data obtained using 1.0% or 1.5% isoflurane were combined because they gave overlapping results. (h, i) Maximal area (h) and full duration at half‐maximum (i) of Ca^2+^ waves recorded from the cochlea of P7–P9 mice under ketamine and xylazine, 2.5% isoflurane and ACP + 1.0%–1.5% isoflurane under constant oxygenation. In panels (g–i) the total number of Ca^2+^ waves identified are: 984 (ketamine and xylazine), 620 (2.5% isoflurane) and 2058 (ACP + 1.0%–1.5% isoflurane). Statistical values in panels (g–i) are from one‐way ANOVA, with Tukey's multiple comparison *post hoc* test. Averaged data are shown as the mean ± SD (red line indicates the median). Abbreviations: ABR, auditory brainstem response; ACP, acepromazine; d*F*/*F*_0_, relative change of fluorescence intensity compared to baseline; IHC, inner hair cell; Iso, isoflurane; Ket, ketamine; P, postnatal day; ROI, region of interest; SCs, supporting cells; Xyl, xylazine.

**FIGURE 5:**
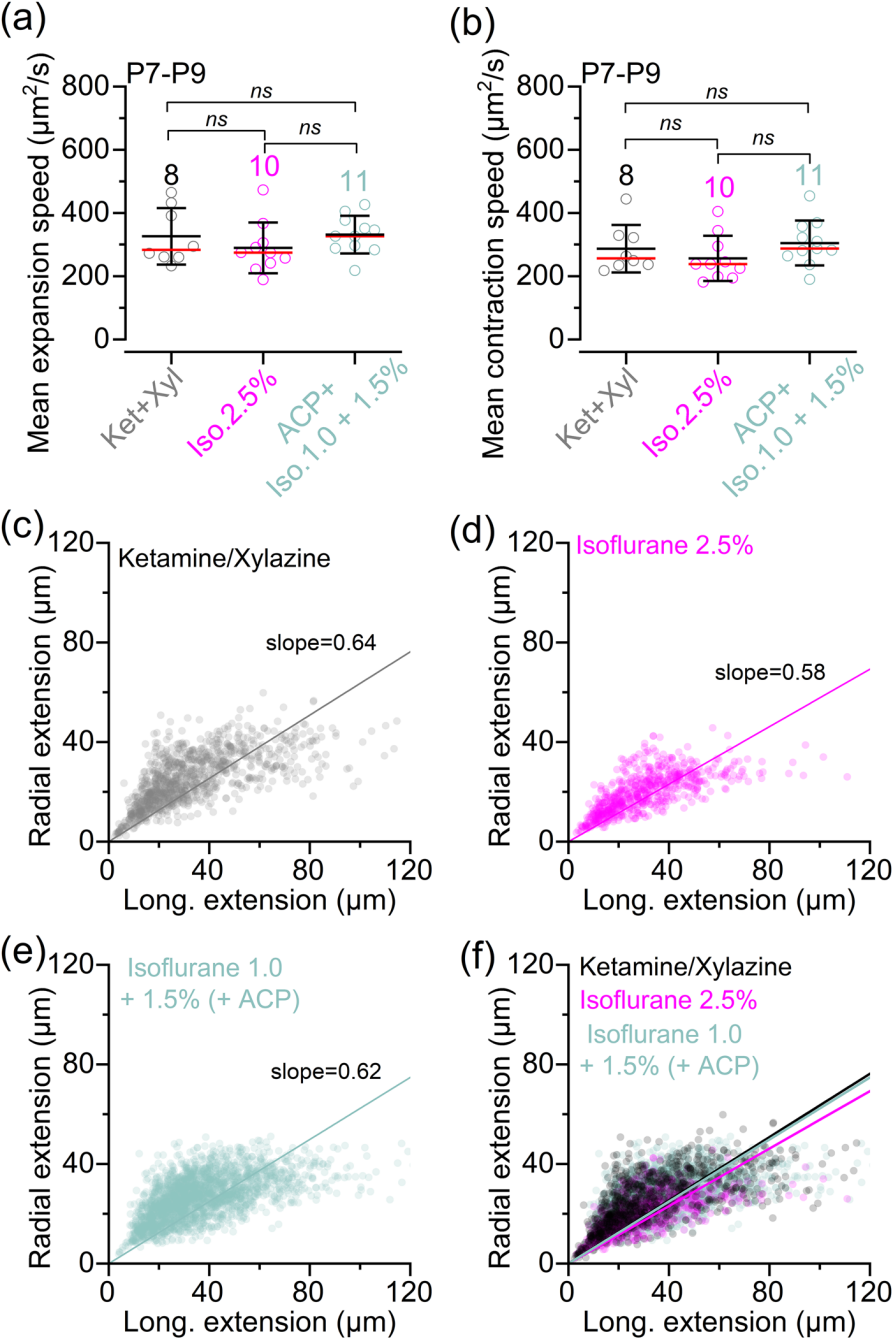
Calcium waves dynamics in anaesthetized pre‐hearing mice. (a, b) Mean Ca^2+^ wave expansion (a) and contraction (b) speed as a function of anaesthetic regime. The number of mice used is: 8 (ketamine and xylazine), 10 (2.5% isoflurane) and 11 (ACP + 1.0‐1.5% isoflurane). Averaged data are shown as mean ± SD (red line indicates the median). Statistical values are from one‐way ANOVA, with Tukey's multiple comparison *post hoc* test. (c–f) Scatterplot showing the relationship between longitudinal and radial extension of all individual Ca^2+^ waves, measured at their maximal expansion when using ketamine and xylazine [(c) 8 mice], 2.5% isoflurane [(d) 10 mice] and ACP + 1.0%–1.5% isoflurane [(e) 11 mice] under constant oxygenation. (f) The three superimposed scatterplots depicted in (c–e). Continuous lines describe a linear fit to the data. Statistical comparisons of the linear fits between the different anaesthetic conditions were performed using a pairwise comparison of the estimated slope coefficients, using Student's two‐tailed *t*‐tests, considering the estimated covariance matrix. The *p*‐values were corrected for multiple comparisons using Bonferroni correction: ketamine and xylazine versus 2.5% isoflurane (*p *= 0.0002); ketamine and xylazine versus 1.0%–1.5% isoflurane (*p *= 0.6498); and 2.5% isoflurane versus 1.0%–1.5% isoflurane (*p *= 0.0015). Abbreviations: ABR, auditory brainstem response; ACP, acepromazine; d*F*/*F*_0_, relative change of fluorescence intensity compared to baseline; Iso, isoflurane; Ket, ketamine; P, postnatal day; Xyl, xylazine.

Using live mice expressing GCaMP6 in the supporting cells (*GCaMP6f* ^fl/fl^ *Pax2‐Cre* ^+/−^), we found that Ca^2+^ waves occurred spontaneously irrespective of the anaesthetic used (Figure 4a–f). The frequency of the Ca^2+^ waves was significantly increased with ketamine–xylazine compared with 2.5% isoflurane (Figure 4g). Although the average maximum area of the Ca^2+^ waves was also significantly reduced in 2.5% isoflurane compared with the other two anaesthetic regimes (Figure 4h), the duration of individual Ca^2+^ waves was unaffected by the anaesthetic used (Figure 4i). Both the mean expansion and contraction speed of the Ca^2+^ waves were comparable across the different experimental conditions (Figure 5a, b). Finally, we found that the shape of Ca^2+^ waves in the cochlea of P7–P9 live mice was similar under all three anaesthetic regimes, with waves preferentially expanding in the longitudinal direction (i.e. along the coil axis of the cochlea; Figure 5c–f).

### 3.6 Calcium transients in the spiral ganglion neuron afferent terminals onto the IHCs

The IHCs are the primary target of type I SGNs (Johnson et al., 2019). During pre‐hearing stages of development, the cochlear endings of SGNs are highly branched and make multiple synaptic contacts with several IHCs (Pujol et al., 1998). As a consequence of pruning during cochlear maturation, SGN terminals form only one‐to‐one axosomatic contact with a given IHC in post‐hearing mice (Liberman & Liberman, 2016; Meyer et al., 2009). The refinement of these synapses, including afferent terminal survival, has been shown to depend upon IHC activity (Lee et al., 2021; Sun et al., 2018; Zhang‐Hooks et al., 2016). The activation of postsynaptic SGN terminals depends on the release of glutamate from IHC synaptic vesicles, which is a Ca^2+^‐dependent process requiring the Ca^2+^ sensor otoferlin (Roux et al., 2006).

**FIGURE 6:**
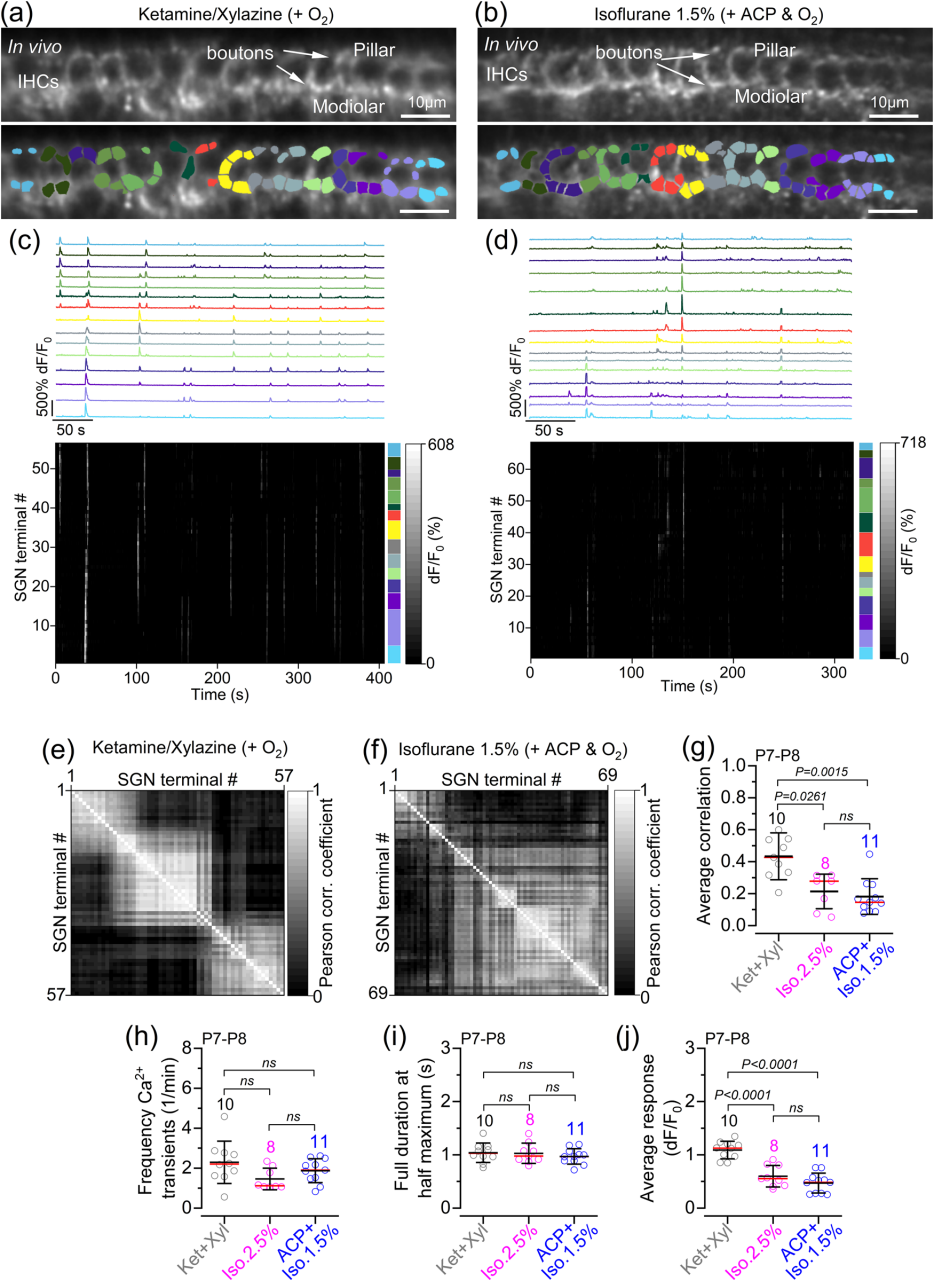
Spontaneous activity in postsynaptic afferent terminals in live pre‐hearing mice under different anaesthetic regimes. (a, b) Average intensity projection displaying GCaMP6 expression in vivo from P8 mice (top; *GCaMP6f* ^fl/fl^ *NeuroD‐Cre* ^+/−^) and segmentation mask highlighting ROIs (bottom) under anaethesia with ketamine and xylazine (a) or 1.5% isoflurane + ACP (b). ROIs were used to identify SGN terminals colour‐matched to their associated IHC. (c, d) Fluorescence signals from individual ROIs in panels (a, b), respectively, shown as average traces per IHC (top panels) and individually (bottom panels: rasterplot; number of SGN terminals: 57 for ketamine and xylazine; 69 for 1.5% isoflurane + ACP). Colour labels on the right of the rasterplot indicate the SGN terminals belonging to the colour‐matched SGNs in the above panels and in panels (a, b), respectively. (e, f) Correlation matrices computed from the in vivo recordings in the presence of ketamine and xylazine or 1.5% isoflurane + ACP shown in panels (c, d), respectively. Each matrix element represents the Pearson correlation coefficient of one pair of SGN terminals. (g) Average correlation coefficient among SGN terminal Ca^2+^ signals recorded in vivo from P7–P9 *GCaMP6f* ^fl/fl^ *NeuroD‐Cre* ^+/−^ mice. (h) Average Ca^2+^ transient frequency in SGN terminals per IHC as a function of anaesthetic regimes, averaged per mouse. (i) Distribution of the full duration at half‐maximum of Ca^2+^ transients from SGN terminals recorded under the three anaesthetic regimes. (j) Average Ca^2+^ signal responses in the SGN terminals. Average data: mean ± SD (red lines indicate the median). The number of mice is shown above the data. Statistical values in panels (g–j) are from one‐way ANOVA, with Tukey's multiple comparison *post hoc* test. Abbreviations: ABR, auditory brainstem response; ACP, acepromazine IHC, inner hair cell; Iso, isoflurane; Ket, ketamine; P, postnatal day; ROI, region of interest; SGN, spiral ganglion neuron; Xyl, xylazine.

Calcium transients in the afferent terminals around the IHCs were investigated in P7–P8 live mice expressing GCaMP6f in the SGNs (*GCaMP6f* ^fl/fl^ *NeuroD1‐Cre* ^+/−^). SGN synaptic terminals were identified as bright hotspots around the IHC basolateral membrane, segmented using a semi‐automated approach (see Materials and Methods). At the image focal plane used, between one and eight hotspots were identified per IHC (average of 3.3 SGN terminals per IHC). Spontaneous Ca^2+^ transients in these afferent terminals were evident on both the modiolar (towards the cochlear nerve) and pillar (towards the outer hair cells) sides of pre‐hearing IHCs (Figure 6a, b). Similar to the Ca^2+^ transients in developing IHCs (Figure 3), we found Ca^2+^ transients in SGN terminals spanning several IHCs and causing the coordinated activation of several afferent terminals in the presence of both ketamine‐based or isoflurane‐based anaesthetics (Figure 6c, d; for 2.5% isoflurane, see De Faveri et al., 2025). This resulted in a high degree of correlation between the activity among SGN terminals both around individual and across several IHCs (Figure 6e–g). When the activity of SGN terminals associated with the same IHC was averaged, we found that the frequency of Ca^2+^ transients and their full duration at half‐maximum did not change significantly across mice under the different anaesthetic regimes (Figure 6h). This agrees with the frequency of Ca^2+^ transients in IHCs (Figure 3i), which drive activity in the SGN terminals. We also found that under ketamine–xylazine anaesthesia, the distribution of Ca^2+^ transient amplitudes was shifted towards higher values compared with isoflurane‐based anaesthetic regimes (Figure 6j). These findings suggest that although the overall frequency and duration of spontaneous activity in SGN terminals appear similar across anaesthetic regimes, ketamine–xylazine might be associated with more robust postsynaptic responses compared with isoflurane‐based anaesthesia.

## 4 DISCUSSION

Here, we investigated the effect of different anaesthetic regimes on the physiology of the pre‐hearing cochlea in live mice. We also assessed the influence of anaesthesia on auditory processing in post‐hearing mice and monitored key physiological parameters, such as heart rate, arterial oxygen saturation and breathing rate. We found that spontaneous Ca^2+^ dynamics in the IHCs, supporting cells and SGN terminals from live pre‐hearing mice were largely preserved between the injectable ketamine–xylazine and the inhalant isoflurane. However, we concluded that low concentrations of isoflurane with the pre‐anaesthetic sedative ACP, rather than injectable anaesthetic, was preferred for mouse pups because it provided a more stable depth of anaesthesia throughout the experiment.

### 4.1 Anaesthesia in neonatal rodents

Performing surgical procedures under deep anaesthesia in neonatal mice is known to be challenging, mainly owing to the difficulties in balancing effectiveness and safety for the still developing animal. Given that neonatal pups are more susceptible to anaesthetics than adult mice, they are more strongly affected by side effects, such as respiratory and cardiovascular depression (Navarro et al., 2021). One of the most common injectable anaesthesia protocols for rodents includes a mixture of ketamine and the sedative xylazine, which has been shown primarily to cause cardiovascular abnormality (Arras et al., 2001; Tsukamoto et al., 2015). The difficulties associated with reaching a safety balance between a surgical plane of anaesthesia and maintaining neonatal rodents alive led to the conclusion that ketamine–xylazine is generally not recommended as the primary choice of anaesthetic for pups (Danneman & Mandrell, 1997; Navarro et al., 2021; Tsukamoto et al., 2017). Furthermore, with injectable anaesthesia, neonatal rodents require a continuous delivery of O_2_, the depth of anaesthesia must be monitored continuously, because it is unpredictable, and the anaesthetics wear off over time. An alternative to injectable anaesthetics is the inhalant isoflurane, which provides a safe and effective approach to achieve a surgical plane of anaesthesia (Danneman & Mandrell, 1997; Huss et al., 2016; Navarro et al., 2021; Tsukamoto et al., 2017).

### 4.2 In vivo cochlear function in anaesthetized mice

It is well established that anaesthetics act by affecting neurotransmission and thus physiological responses (e.g. Hao et al., 2020; Platholi & Hemmings, 2022). Ketamine is a glutamatergic NMDA receptor antagonist, whereas isoflurane affects both NMDA and AMPA receptors (Barrett et al., 2020; Carlà & Moroni, 1992; Petrenko et al., 2014). In mice, both anaesthetics have been shown to affect hearing function, albeit to different degrees. Ketamine is the preferred anaesthetic to measure hearing function because it leads to relatively small increases in both ABR thresholds and ABR‐peak latencies in comparison to awake mice (Van Looij et al., 2004). Isoflurane, is known to greatly affect hearing thresholds in mice (Cederholm et al., 2012; Ruebhausen et al., 2012; Verdoodt et al., 2021), which was further validated by our findings. However, we found that lowering the concentration of isoflurane to 1.0%–1.5%, with the prior injection of the pre‐anaesthetic sedative agent ACP (Arras et al., 2001), largely mitigated the side effects of this anaesthetic on ABR recordings in adult mice.

Given that the physiology and pharmacology of anaesthetics vary significantly between adult and neonatal mice (Navarro et al., 2021), and ABR cannot be measured in pre‐hearing mice, we assessed the possible pharmacodynamic side effects of both ketamine and isoflurane regimes in pups by measuring cochlear function in vivo and clinical vital signs in anaesthetized mice. We found that Ca^2+^ signal dynamics in IHCs and SGN terminals from mice anaesthetized with isoflurane were comparable to those treated with the injectable anaesthetic ketamine. Spontaneous Ca^2+^ waves were also observed in the supporting cells from live mice treated with all anaesthetic regimes. However, 2.5% isoflurane appeared to produce a slight reduction in the frequency and maximum area of the Ca^2+^ waves in comparison to ketamine, although all the other characteristics remained unchanged. Within the cochlear partition, we found that the EP, which is crucial for mechanoelectrical transduction in hair cells, was indistinguishable among the anaesthetic regimes used. The most challenging aspect of using the injectable anaesthetic was the difficulty in maintaining a stable depth of anaesthesia and the need of at least one follow‐up injection, which also agrees with recent findings (Navarro et al., 2021; Tsukamoto et al., 2017). Measurement of clinical signs in anaesthetized live mice have highlighted that the heart rate, breathing rate and pulse distension decrease progressively over time in the presence of ketamine, but were more stable with the lower concentration of isoflurane. We concluded that, although some significant differences were observed between the anaesthetic regimes tested, their overall impact on the dynamics of spontaneous activity across cochlear cell types was relatively minor. Based on the above findings, we propose that the use of the pre‐anaesthesia sedative ACP with 1.0%–1.5% isoflurane provides the overall best compromise to investigate cochlear function in vivo in pre‐hearing mice, while maintaining stable vital signs and depth of anaesthesia.

## AUTHOR CONTRIBUTIONS

All authors performed the experiments, helped with the interpretation of the data and contributed to the writing of the paper. Walter Marcotti and Federico Ceriani conceived and coordinated the study. All authors approved the final version of the manuscript and agree to be accountable for all aspects of the work in ensuring that questions related to the accuracy or integrity of any part of the work are appropriately investigated and resolved. All persons designated as authors qualify for authorship, and all those who qualify for authorship are listed.

## CONFLICT OF INTEREST

None declared.

## Data Availability

The data that support the findings of this study are available from the corresponding author upon reasonable request. Code used for the analysis is available at https://github.com/Marcotti‐Lab/invivo‐IHC‐cochlea.
